# Early Outcomes of Repetitive Transcranial Magnetic Stimulation in Complex Clinical Population

**DOI:** 10.1192/j.eurpsy.2023.389

**Published:** 2023-07-19

**Authors:** A. V. Samokhvalov, M. Weber

**Affiliations:** ^1^ASU / CPC, Homewood Health Centre, Guelph; ^2^Department of Psychiatry, University of Toronto, Toronto; ^3^Interventional Psychiatry, Homewood Health Centre, Guelph; ^4^Department of Psychiatry & Behavioural Neurosciences, McMaster University, Hamilton; ^5^Dr. Sam’s Health, Oakville, Canada

## Abstract

**Introduction:**

Repetitive Transcranial Magnetic Stimulation (rTMS) is a new emerging neuromodulation treatment that has been tried for multiple psychiatric conditions [1, 2]. Its major approved application is treatment-resistant depression (TRD) [1]. At the same time there is a perceived potential for its use for other clinical conditions, primarily other mood and anxiety disorders [2]. At Homewood Health Centre we have been using rTMS as an adjunct treatment for patients with TRD and multiple comorbidities.

**Objectives:**

To evaluate the effectiveness and feasibility of rTMS in complex clinical populations.

**Methods:**

Observational study. Quick Inventory of Depressive Symptomatology (QIDS). Generalized Anxiety Disorder Questionnaire (GAD-7). Descriptive statistics.

**Results:**

We have treated 30 patients, 12 women (40%) and 18 men (60%), with average age of 42.0±15.6 years. All patients had a primary diagnosis of major depressive disorder. The standard questionnaires were used to quantify the severity of depressive symptoms (QIDS) and anxiety (GAD-7). The average baseline scores for depression and anxiety were 16.1±4.9 and 15.0±4.4, respectively. The patients received an average of 28.1±5.1 treatments. All patients but one received the full course of treatment as planned. The average end-of-treatment (EoT) scores for severity of depressive symptoms and anxiety were 9.6±6.5 and 7.3±5.3, respectively. The rates of improvement and remission for depressive symptoms were 66.7% and 36.7%, respectively. The rates of improvement and remission for anxiety symptoms were 76.9% and 30.8%, respectively.

**Image:**

**Figure:**
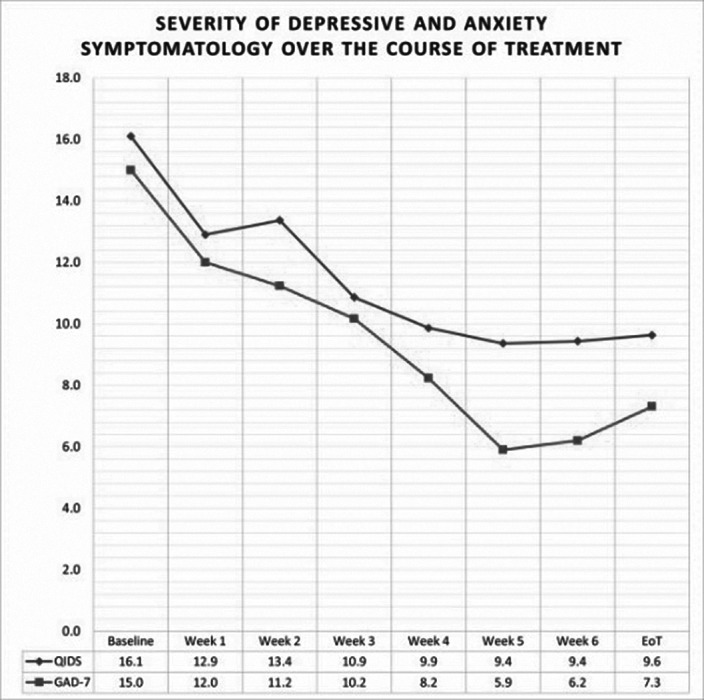

**Conclusions:**

Our data indicate that rTMS provides significant improvement and recovery rates in complex clinical populations and is well-tolerated. While further research is required, we recommend wider implementation of rTMS for treatment of mood and anxiety disorders.

**References:**

1. Brunoni AR, Chaimani A, Moffa AH, Razza LB, Gattaz WF, Daskalakis ZJ, Carvalho AF: Repetitive Transcranial Magnetic Stimulation for the Acute Treatment of Major Depressive Episodes. JAMA Psychiatry 2017, 74(2):143.

2. Somani A, Kar SK: Efficacy of repetitive transcranial magnetic stimulation in treatment-resistant depression: the evidence thus far. Gen Psychiatr 2019, 32(4):e100074.

**Disclosure of Interest:**

None Declared

